# Rapid, Highly-Efficient and Selective Removal of Anionic and Cationic Dyes from Wastewater Using Hollow Polyelectrolyte Microcapsules

**DOI:** 10.3390/molecules28073010

**Published:** 2023-03-28

**Authors:** Zhiqi Zhao, Hongbing Zhou, Xu Han, Lun Han, Zhenzhen Xu, Peng Wang

**Affiliations:** 1 School of Textile and Garment, Anhui Polytechnic University, Wuhu 241000, China; 2 Zhejiang Huaguang Automotive Interior Decoration Co., Ltd., Rui’an 325200, China

**Keywords:** polyelectrolyte microcapsule, dyes removal, water treatment, high adsorption capacity, fast adsorption

## Abstract

Herein, poly (allylamine hydrochloride) (PAH)/ poly (styrene sulfonic acid) sodium salt (PSS) microcapsules of (PAH/PSS)_2_PAH (P2P MCs) and (PAH/PSS)_2_ (P2 MCs) were obtained by a layer-by-layer method. The P2 MCs show high adsorption capacity for Rhodamine B (642.26 mg/g) and methylene blue (909.25 mg/g), with an extremely low equilibrium adsorption time (~20 min). The P2P MCs exhibited high adsorption capacities of reactive orange K-G (ROKG) and direct yellow 5G (DY5G) which were 404.79 and 451.56 mg/g. Adsorption processes of all dyes onto microcapsules were best described by the Langmuir isotherm model and a pseudo-second-order kinetic model. In addition, the P2P MCs loaded with reactive dyes (P2P–ROKG), could further adsorb rhodamine B (RhB) dye, and P2 MCs that had adsorbed cationic MB dyes could also be used for secondary adsorption treatment of direct dye waste-water, respectively. The present work confirmed that P2P and P2 MCs were expected to become an excellent adsorbent in the water treatment industry.

## 1. Introduction

Dyes are applied to textile [1], painting [2], plastics [3] and other industrial fields, resulting in large amounts of dye wastewater. Increasing application of reactive dyes and direct dyes (anionic dye) in the textile dyeing and printing industry is due to the performance of their complete chromatography and the bright color of viscose, cotton and other fabrics [4,5,6]. Therefore, wastewater treatment of reactive and direct dyes has important research significance. Meanwhile, cationic dye applications are increasing due to the rapid pace of industrial and technical developments. These dyes seem to be more toxic than other dyes as cationic dyes can be easily absorbed by human cells and further accumulate in the cytoplasm [7]. Rhodamine B (RhB) and methylene blue (MB) are widely researched in both the textile industry and biological research [8]. Although reactive dyes, the direct dyes, RhB and MB are widely used in production, but there are still questions about the monitoring of these dyes, because their dye wastewater is often discharged into the aquatic environment with improper management.

Once the dye wastewater is discharged into water, it poses a serious threat to the human and ecological environment because it is toxic, non-biodegradable and even carcinogenic [9,10,11]. At the same time, the dye is generally stable to light, heat, oxidation, and is soluble in water. Much research has focused on the photo-degradation of many kinds of dye, but the high cost of photo-catalytic materials limits the wide application of photo-degradation of dye wastewater [12]. Compared with the photo-degradation method, the adsorption method [13,14,15] has the advantages of simple operation [16,17], high efficiency [18] and low cost, etc., which is especially suitable for the treatment of very complex wastewater pollutants, which have been widely studied in wastewater treatment [19,20,21]. Many materials, such as metal–organic frameworks (MOFs) [22] and gel material with voids [23] or functional groups [24] could be applied as adsorbents, but many adsorbents have the disadvantage of low adsorption capacity [25,26]. Based on these concepts, it is of great significance to develop new materials with selectivity and high adsorption capacity. It is worth noting that most industrial wastewater is composed of multiple dyes, and the absorption of dyes from a mixed system has more potential applications than that from a single system.

Many porous materials have been applied to dye adsorption in wastewater [27]. The introduction of the special structure of the adsorbent, such as the interaction with the functional dye groups, can improve the selectivity of the adsorbent. Because of its specific surface area and water stability, microcapsules [28,29,30] have been successfully used for dye adsorption. As an adsorbent material, microcapsules can remove a variety of dyes in wastewater well because of their two advantages: one is to offer adsorption sites; the second is the quasi-spherical hollow structure of microcapsules. However, slow adsorption rates and removal rates hinder the development of their application. Polyelectrolytes such as the wall of microcapsules, have related functional groups which can have certain electrostatic effects with dyes. Because of their porous structure and good adsorption performance, microcapsules can achieve rapid adsorption of various drugs and dyes, and are also used as nano carriers in chemistry, biomedicine, electromagnetics and environmental science. Surface area and surface functional groups are two pivotal parameters to determine the adsorption capacity of anionic and cationic dyes [31,32,33,34,35]. (PAH/PSS)_2_PAH and (PAH/PSS)_2_ microcapsules can selectively remove anionic and cationic dyes based on synergistic effects of porous and hollow structures and electrostatic interactions. The high specific surface area and effective functional groups of (PAH/PSS)_2_PAH and (PAH/PSS)_2_ microcapsules’ material promoted the adsorption of anionic and cationic dyes.

We have synthesized polyelectrolyte microcapsules by a layer-by-layer method at room temperature [36,37]. Hollow polyelectrolyte microcapsules revealed a high surface to volume ratio, and significant loading capacity. To evaluate adsorption selectivity of polyelectrolyte microcapsules towards organic dyes, new hollow (PAH/PSS)_2_PAH as P2P and (PAH/PSS)_2_ as P2 microcapsules were constructed by coating PAH and PSS onto the surface of CaCO_3_ to generate core@shell structured CaCO_3_@(PAH/PSS)_2_PAH and CaCO_3_@(PAH/PSS)_2_ and then removing the CaCO_3_ templates. The (PAH/PSS)_2_PAH (P2P MCs) and (PAH/PSS)_2_ (P2 MCs) were applied to remove anionic and cationic dyes. The P2 MCs show high adsorption capacity for Rhodamine B and methylene blue, with an extremely low equilibrium adsorption time (~20 min). The P2P MCs exhibited high adsorption capacities for reactive orange K-G (ROKG) and direct yellow 5G (DY5G). The P2P MCs loaded with reactive dyes (P2P–ROKG) could further adsorb RhB, and P2 MCs that had adsorbed cationic MB dyes could also be used for the secondary adsorption treatment of direct dye waste-water, respectively. At the same time, we investigated effects of initial dye concentration, time and solution pH on dye adsorption. In addition, we evaluated adsorption isotherms, adsorption kinetics and possible selective adsorption mechanisms of P2P and P2 microcapsules for four dyes. These studies proved that P2P and P2 microcapsules are promising adsorbents and can be used to treat simulated dye wastewater containing cationic and anionic dyes.

## 2. Results and Discussion

### 2.1. Microstructure and Morphology of P2P and P2 Microcapsules

**Figure 1:**
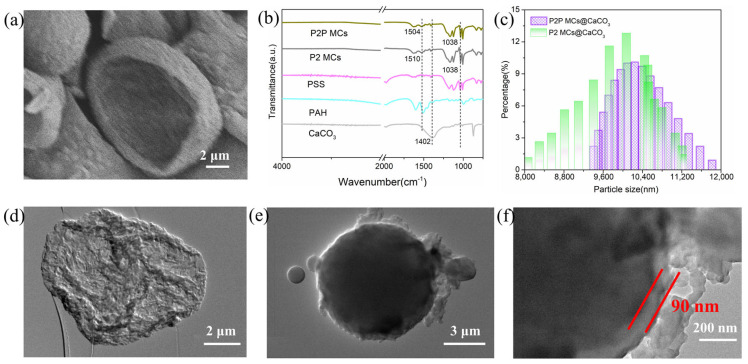
(**a**) SEM images of the microcapsules, (**b**) FTIR spectra of PAH, PSS, CaCO_3_, P2 and P2P microcapsules, (**c**) Size distribution of the microcapsules, (**d**–**f**) TEM of the microcapsules.

Mono-disperse PSS doped CaCO_3_ particles with a diameter of 8~12 μm were used as a sacrificial template (Figure S1). PAH and PSS were employed to form (PAH/PSS)_2_PAH and (PAH/PSS)_2_ microcapsules through a layer-by-layer (LBL) method. CaCO_3_ particles were etched by EDTA to yield the hollow space for dye adsorption. Scheme S1 shows the schematic process of preparing polyelectrolyte P2P and P2 microcapsules. The morphology and structure of as-synthesized hollow P2P and P2 microcapsules are revealed by SEM (Figure 1a) and TEM (Figure 1d). As shown in Figure 1e and Figure S2, P2P and P2 microcapsules exhibited spherical morphology. Spherical adsorbents have more advantages in diffusion and transport than other structural adsorbents [38]. In addition, TEM images indicated that the microcapsules became folded and wrinkled after being dried (Figure 1a) and a had mean diameter of 8~12 μm after size distribution testing, and the shell thickness was evaluated to be about 90 nm (as the red lines show), as shown in Figure 1c,f. The hollow structure of microcapsules with many adsorption sites and spaces provided for adsorption of dye molecules and so P2P and P2 microcapsules have higher adsorption capacity. FTIR spectra of PAH, PSS, CaCO_3_, P2 and P2P microcapsules are displayed in Figure 1b. FTIR spectra of two microcapsules were very similar. Whereas in the spectrum of P2P and P2 microcapsules the aromatic ring stretch signals from the PAH/PSS complex were found at 1510 cm^−1^ and the band of PSS was assigned at 1038 cm^−1^ due to S–O stretching vibration deformation [39,40] (Figure 1c). As shown in Figure S3, the thermochemical behaviors of P2P and P2 microcapsules were demonstrated by TG-DTG, which further detected the components of the composite microcapsules, as shown in Figure 1d. DTG and TG curves of two kinds of P2P and P2 microcapsules are very similar. The weight loss process could contain three stages, including loss of absorbed water, combustion of the wall material of the microcapsules and decomposition of a little residual EDTA. At the first stage, there was a small exothermic peak and 10 ± 1% mass loss at 59–212 °C. A significant endothermic peak at 367–415 °C and 75 ± 5% weight loss was attributed to the thermal decompositions of PAH and PSS [41]. CaCO_3_ had a big peak in the range of 740–780 °C, but there was no corresponding peak in the corresponding positions of the three microcapsules, which indicates that the CaCO_3_ template in the microcapsules has been removed, and this result is also consistent with the FTIR and SEM analysis [42,43,44].

### 2.2. Adsorption of Dyes

**Figure 2:**
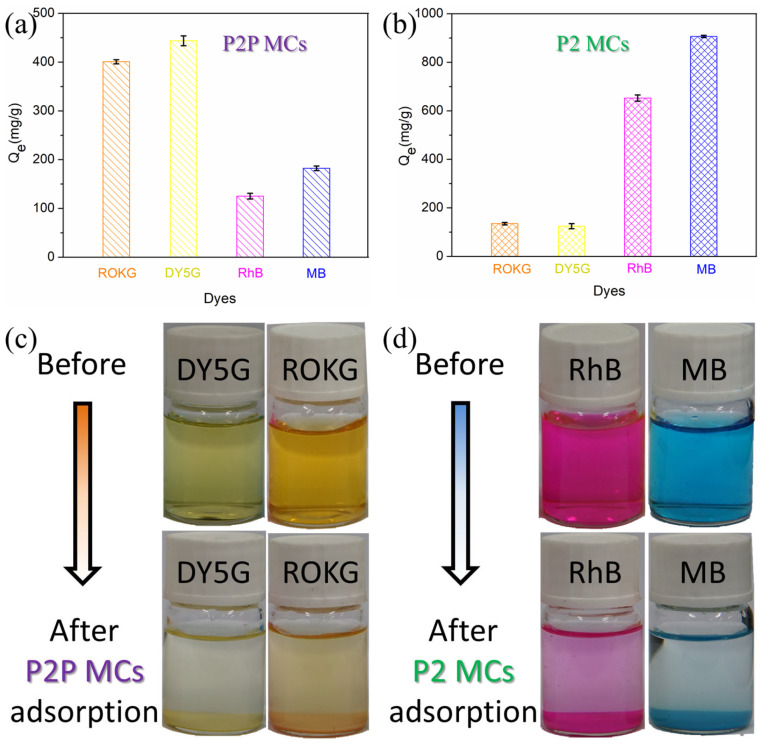
Equilibrium adsorption capacities of the hollow P2P (**a**) and P2 (**b**) microcapsules towards four dyes (ROKG, DY5G, RhB and MB). Digital photos of the four dyes in aqueous solution before and after adsorption by the hollow P2P(ROKG and DY5G) (**c**) and P2(NR, RhB and MB) (**d**) microcapsules under the same conditions.

The selective adsorption of dyes is of great significance because it not only facilitates the adsorption of dyes, but also facilitates the selective separation of dyes from wastewater. Microcapsules confer selective adsorption properties by interacting with specific types of dyes (anions or cations). The surface of P2P microcapsules contain many cationic functional groups (–NH_3_^+^), while the surface of P2 microcapsules contain anionic functional groups (–SO_3_^−^), so that dyes with opposite electrical properties can be adsorbed through electrostatic interaction [45]. To evaluate the adsorption properties of P2 and P2P microcapsules for dyes, four dyes with different charges were selected for the adsorption study, including ROKG and DY5G, for anionic dyes, MB and RhB for cationic dyes. The equilibrium adsorption capacity (Q_e_) of hollow P2P microcapsules to AOCK, DY5K, RhB and MB dyes were 409.80, 451.64, 130.89, and 185.43 mg/g, respectively, which are different from P2 microcapsules (131.17, 111.74, 642.28 and 909.19 mg/g), as plotted in Figure 2 (initial dye concentration: 3000 mg/L) at 25 °C for 5 h.

The hollow P2(MB and RhB) and P2P(AOKG and DY5G) microcapsules were mixed with the dye solution, respectively, and then digital photos were taken to compare the color change of the dye solution before and after adsorption, as shown in Figure 2c,d. After adsorption, the solution color of dyes such as AOKG, DY5G, RhB, MB and NR were obviously close to colorless, which shows that P2P and P2 microcapsules have good adsorption capacity for these dyes.

**Figure 3:**
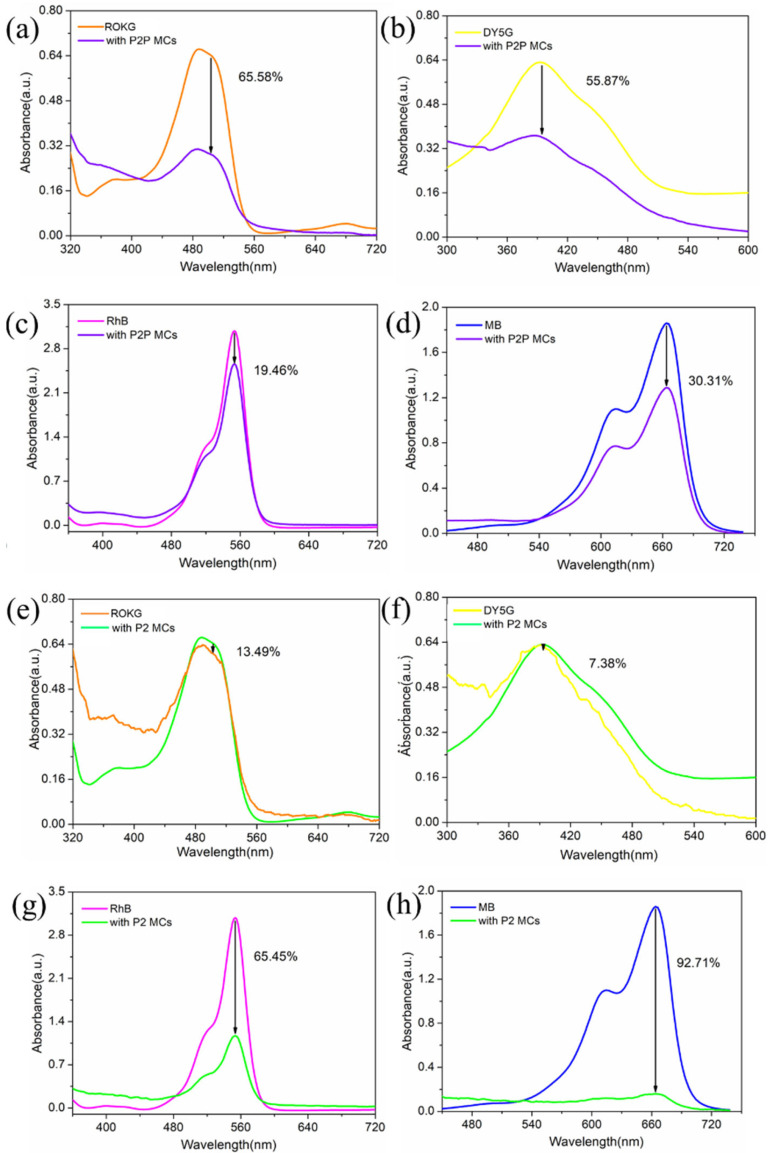
Changes of UV–Vis spectra and removal efficiency of ROKG (**a**,**e**), DY5G (**b**,**f**), RhB (**c**,**g**) and MB (**d**,**h**) solutions (3000 mg/L) with the addition of P2P and P2 (both 0.2 mg/mL), respectively.

In Figure 3, removal efficiencies (R) of P2P microcapsules to ROKG and DY5G are 65.58% and 55.87%, respectively (Figure 3a,b). The R of cationic dyes RhB and MB are 19.46% and 30.31%, respectively (Figure 3c,d). The hollow P2P microcapsule has many R–NH_3_^+^ groups on the surface of the microcapsule wall, which leads to positive charges on the microcapsules. Due to the electronegativity of ROKG and DY5G dyes, hollow P2P microcapsules easily form strong electrostatic interactions with them. However, electrostatic repulsion between adsorbent and cationic dyes results in a lower R of hollow P2P microcapsules to two dyes. Oppositely, as shown in Figure 3e–h, for P2 microcapsules, the removal efficiencies are 13.49% (ROKG) and 7.38% (DY5G) for anionic dyes, and the data can increase to 65.45% and 92.71% for cationic dyes of RhB and MB by P2 microcapsule adsorption. Electrostatic interaction is the main reason for the high adsorption capacity of cationic dyes. The R of anionic dyes is relatively low or even negligible. P2 microcapsules contain a large amount of negatively charged groups (–SO_3_^−^). Similarly, the adsorption of hollow P2 microcapsules for dyes is highly dependent on the electrostatic interaction of –SO_3_^−^ groups and dye molecules, so it has better selective adsorption capacity for cationic dyes.

#### 2.2.1. Effect of Initial Dye Concentrations on Dye Adsorption

**Figure 4:**
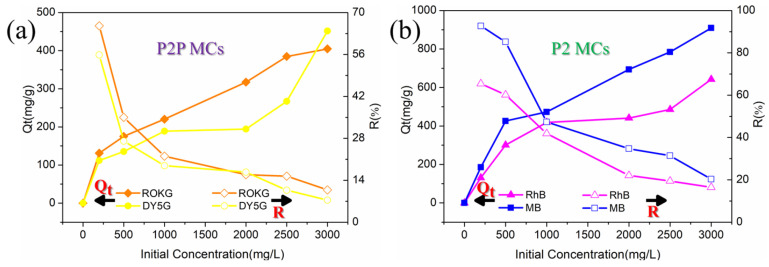
Adsorption effects of initial concentrations of anionic dyes (ROKG and DY5G) by P2P MCs (**a**) and cationic dyes (RhB and MB) by P2 MCs (**b**), 2 mg of adsorbent was added to the aqueous solution of ROKG, DY5G, RhB and MB (initial dye concentration: 200–3000 mg/L) at 25 °C for 5 h, respectively.

As displayed in Figure 4 the adsorption capacity of microcapsules for dyes increases with the increase in dye concentration (initial dye concentration: 200–3000 mg/L) at 25 °C for 5 h. On the contrary, the dye removal rate gradually decreases with the increase in dye concentration. This is mainly because the adsorption sites of microcapsules for dye molecules are limited, and the intermolecular competition of high concentration dye solutions is more intense [46]. P2P and P2 microcapsules can adsorb dye more effectively at higher dye concentrations.

#### 2.2.2. Effect of Time of Dye on Adsorption

**Figure 5:**
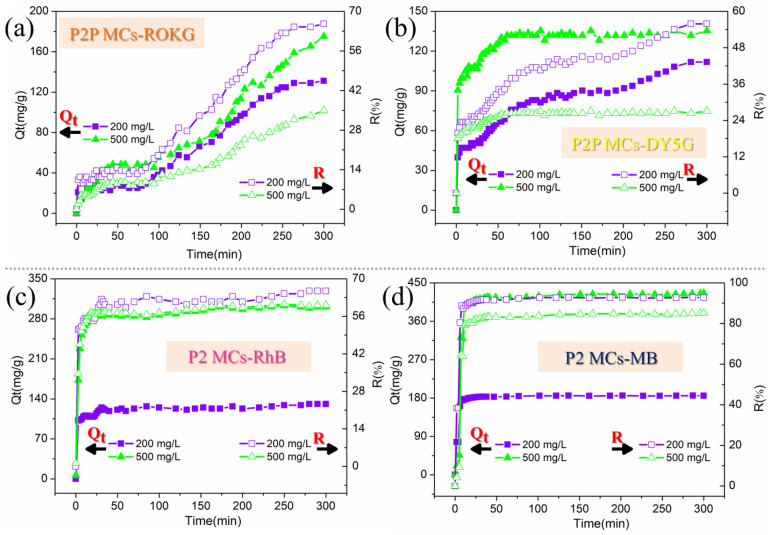
Effects of time on adsorption of ROKG (**a**) and DY5G (**b**) on hollow P2P microcapsules, and RhB (**c**) and MB (**d**) on hollow P2 microcapsules, 2 mg of adsorbent was added into the solutions of ROKG, DY5G, RhB, and MB (initial dye concentration: 200–500 mg/L) at 25 °C for 5 h, respectively.

Figure 5 shows the test results of adsorption performance of P2 and P2P microcapsules on anionic and cationic dyes at different times, initial dye concentration: 200–500 mg/L at 25 °C for 5 h. The experimental results showed that adsorption of RhB and MB on P2 microcapsules’ adsorption capacity increases with the increase in the two dyes’ concentration. Compared with adsorption of the anionic dyes (ROKG and DY5G) by P2P microcapsules, the adsorption of P2 microcapsules were faster and more stable, and increased rapidly from 2 min to 20 min; when the adsorption time t was more than 20 min, the adsorption capacity did not change significantly, it can be considered that the adsorption balance of methylene blue dye on microcapsules was reached at about 20 min. The charge characteristics and the hollow structure of the microcapsules provide sufficient conditions for dye adsorption. This phenomenon can be explained by the increase in dye concentration increasing the driving force of the concentration gradient. Therefore, when the adsorption capacity with dye reaches equilibrium, dye molecules cannot continue to be adsorbed by microcapsules. The adsorption behavior of P2 microcapsules was better under conditions of higher cation concentration. The adsorption behavior of P2 microcapsules for cationic dye was better than that of P2P microcapsules for anionic dye, which may be due to the existence of PSS in the microencapsulated cavity, which has a certain degree of electronegativity. At a lower initial concentration (200 mg/L), dye adsorption efficiency is higher than that at a higher initial concentration (500 mg/L). When the dye concentration is high, the proportion of available adsorption sites decreases [47,48].

#### 2.2.3. Effect of pH on Dye Adsorption

**Figure 6:**
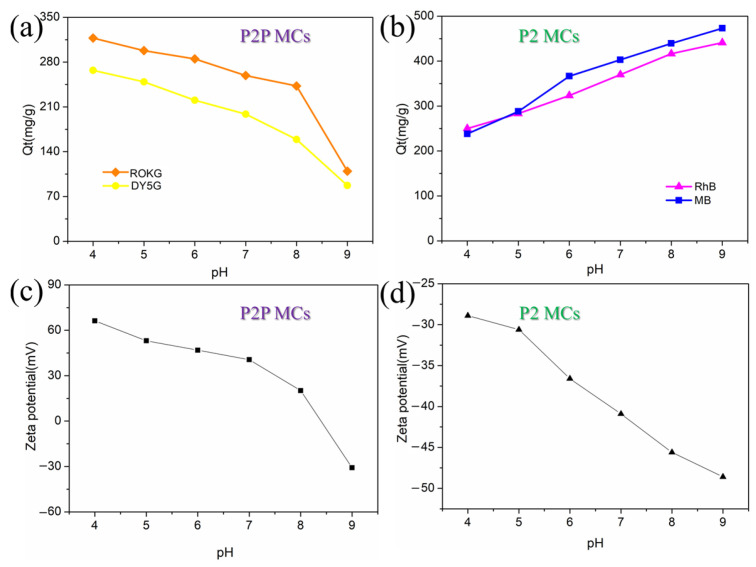
Effect of pH on adsorption capacity of ROKG and DY5G by P2P MCs (**a**), and RhB and MB by P2 MCs (**b**). Zeta potential of P2P MCs(**c**) and P2 MCs (**d**) at different pH values.

Under acidic conditions with a pH of 4–7, electrostatic interaction between protonated functional groups (–NH_3_^+^) of P2P microcapsules and negatively charged anionic dyes (ROKG and DY5G) increases. If the dye concentration was 2500 mg/L, the amount of adsorbent was 2 mg at 25 °C for 5 h, as shown in Figure 6a. Under alkaline conditions, electrostatic repulsion occurs between deprotonated functional groups of P2P microcapsules and negatively charged anionic dyes [49]. In Figure 6b, adsorption capacity of P2 microcapsules to dyes increases with increasing pH value. The maximum adsorption capacity is reached at pH = 9. In an acidic environment, the functional groups of P2 microcapsules undergo protonation, and electrostatic repulsion between P2 microcapsules and cationic dyes increases. Under alkaline conditions, the functional groups of P2 microcapsules become more deprotonated, and the adsorption capacity of P2 microcapsules for RhB and MB (cationic dyes) is much higher [46].

To determine the point of zero charge of P2P MCs microcapsules, the zeta potential was measured in solutions with different pH values of 4–9, as shown in Figure 6c. The zeta potential of P2P MCs microcapsules were negative at a pH of 9. As the pH decreases, the zeta potential first becomes positive, and then increases as the pH decreases. The dissociated R–NH_3_^+^ increases the adsorption amount of anionic dyes on the microcapsules. As shown in Figure 6d, the zeta potential of P2 MCs decreases with the increase in pH value [50,51]. This is due to the increased dissociation of the PSS sulfonic acid group, and the gradual increase in the adsorption capacity of P2 MCs to cationic dyes.

### 2.3. Adsorption Kinetic Study

**Figure 7:**
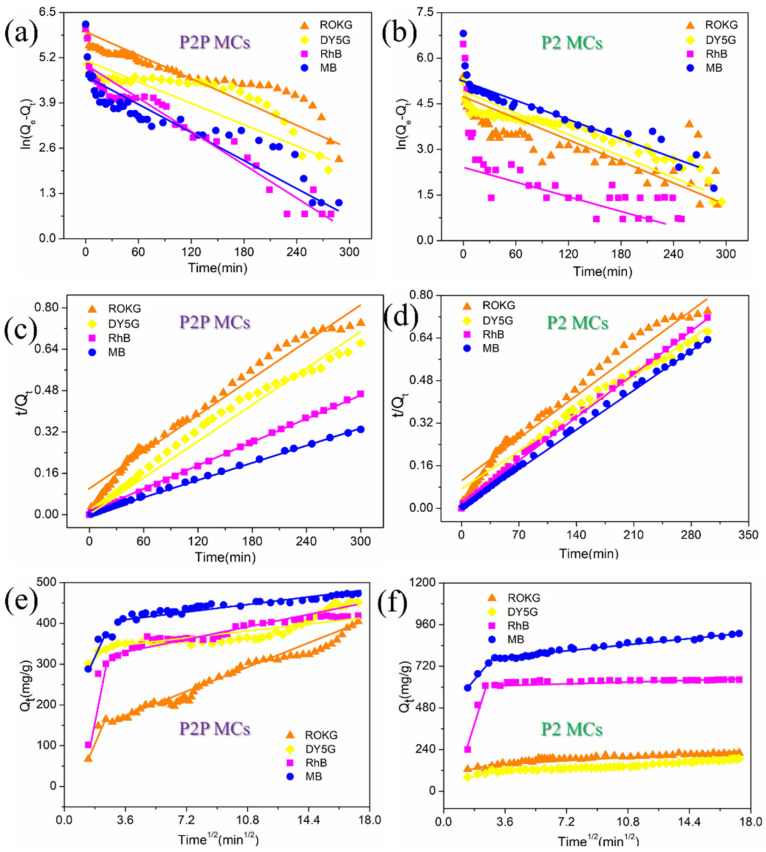
Pseudo-first-order kinetic model for adsorption of the ROKG, DY5G, RhB and MB dyes onto the P2P microcapsules (**a**) and P2 microcapsules (**b**), pseudo-second-order kinetic model for adsorption of ROKG, DY5G, RhB and MB dyes onto the P2P microcapsules (**c**) and P2 microcapsules (**d**), intraparticle diffusion model for adsorption of four dyes on P2P microcapsules (**e**) and P2 microcapsules (**f**).

Benefiting from porous and hollow structures, high specific surface areas as well as good thermal stability, both P2 and P2P microcapsules are excellent adsorbent candidates for anionic and cationic dyes. To reveal the adsorption property of ROKG, DY5G, RhB and MB dyes on P2 and P2P microcapsules, the dye solution was prepared with an initial concentration of 1000 and 3000 mg/L, 2 mg of the adsorbent microcapsule was added, 10 μL supernatant after a certain time was used to determine the absorbance, and the absorbance measured at different times was used to calculate the adsorption amount. The pseudo-first-order and pseudo-second-order kinetic models were employed to fit the adsorption kinetics data, respectively (Figure 7a–d).

The pseudo-first-order and pseudo-second-order kinetic models can be described by Equations (1) and (2) [52]:

(1) $$\ln\left(Q_{e}-Q_{t}\right)=lnQ_{e}-k_{1}t$$

(2) $$\frac{t}{Q_{t}}=\frac{1}{k_{2}Q_{e}^{2}}+\frac{1}{Q_{e}}t$$

where *Q_e_* and *Q_t_* represent the adsorption capacity of microcapsules at the equilibrium time and *t* time (mg/g), *k*_1_ (min^−1^) and *k*_2_ (g·mg^−1^·min^−1^) represent the rate constants in quasi first order and quasi second order kinetic models, respectively.

For P2P and P2 microcapsules, pseudo-second-order equation fitted with a pseudo high correlation coefficient value R^2^ = 0.9771, which means that the quasi second-order kinetic model is more suitable to describe the adsorption process of P2P and P2 microcapsules than the quasi first-order kinetic model and was very close to *Q_e_*. Results can be found in Table 1 and Figure 7c,d. The pseudo-secondary kinetic model was based on chemical adsorption control [53]. 

The adsorption kinetics of the three dyes on the microcapsules was simulated by Weber’s model [54]. The model can be expressed in linear form as Equation (3):

(3) Qt=kit12+c

where *k_i_* (mg·g^−1^ min^−1/2^) is the intraparticle diffusion rate constant and *c* (mg/g) is a constant involving the thickness and boundary layer.

Figure 7e,f shows the linear diagram of the diffusion process for four dyes (ROKG, DY5G, RhB and MB) in P2 and P2P microcapsules. The diffusion processes were divided into two steps [55]. The first stage is diffusion of dye molecules from the solution to the surface of the microcapsules (membrane diffusion). The second stage is the internal diffusion of the microcapsules, which is caused by a large number of gaps in the wall structure of the polyelectrolyte multilayered microcapsules. As shown in Figure 7e,f the slope of the film diffusion stage (*k*_*i*1_) is larger than that of the internal diffusion stage (*k_i1_*), indicating that the internal diffusion stage (*k*_*i*2_) is a gradual process, which conforms to Weber’s particle internal diffusion model (Table 1 and Table 2). In addition, the higher constant C indicates that the mass transfer of external dye on microcapsules at an early stage of adsorption is significant in the adsorption process. In addition, larger *R*_*i*1_^2^ and *R*_*i*2_^2^ show that the Weber’s particle internal diffusion model has wide applicability in the study of dye adsorption processes of microcapsules.

### 2.4. Adsorption Isotherm

**Figure 8:**
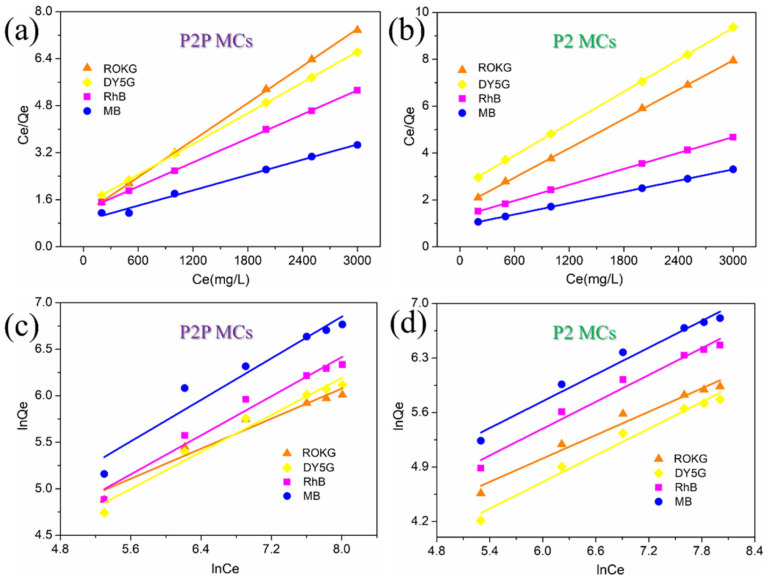
The Langmuir model for the adsorption of ROKG, DY5G, RhB and MB dyes onto the P2P (**a**) and P2 (**b**) microcapsules, the Freundlich model for four representative dyes’ adsorption on P2P (**c**) and P2 (**d**) microcapsules.

The maximum adsorption capacities (*Q_max_*) of P2P and P2 microcapsules for two anionic dyes ROKG, DY5G and two cationic dyes RhB, MB have been investigated. For researching the adsorption mechanism of P2P and P2 microcapsules on dyes, the Langmuir equation [56] (Formula (4)) and Freundlich equation [57] (Formula (5)) were used to fit experimental data of the four dyes, as shown in the following equations:

(4) $$\frac{C_{e}}{Q_{e}}=\frac{1}{K_{L}Q_{max}}+\frac{C_{e}}{Q_{max}}$$

(5) $$lnQ_{e}=lnK_{F}+\frac{1}{n}lnC_{e}$$

where *C_e_* (mg/L) and *Q_e_* (mg/g) represent dye concentration at equilibrium and dye adsorption capacity of microcapsules. *K_L_* (L/mg) and *Q_max_*, respectively, represent adsorption constant and maximum adsorption capacity in the Langmuir adsorption model. *C_e_* is the abscissa and *C_e_/Q_e_* is the ordinate. *K_L_* and *Q_max_* can be calculated by the straight-line slope and intercept obtained by fitting (Figure 8). *K_F_* ((mg/g) (L/mg)^1/n^) is the adsorption constant in the Freundlich model, and n is the heterogeneity factor [58].

Linear fitting results of Langmuir and Freundlich models towards four anionic dyes (ROKG, DY5G, RhB and MB) are displayed in Figure 8a–d, respectively. The related correlation coefficients (R^2^) and equilibrium parameters are listed in Table 3 and Table 4. For P2P microcapsules, the R^2^ (0.9492, 0.9660, 0.9605, 0.9309) of the Freundlich model for dyes (ROKG, DY5G, RhB and MB) was lower than that of the Langmuir model (0.9979, 0.9994, 0.9989, 0.9891), which indicates that the Langmuir model was more suitable for describing the adsorption of dye by adsorbent microcapsules. Similarly, the Langmuir model can better fit adsorption data than the Freundlich isotherm model, and the regression coefficient of P2 microcapsules was higher (R^2^ > 0.99). K_L_ ranges from 0 to 1, indicating that two microcapsules were beneficial for the adsorption of four dyes [52].

### 2.5. Adsorption Mechanisms

**Figure 9:**
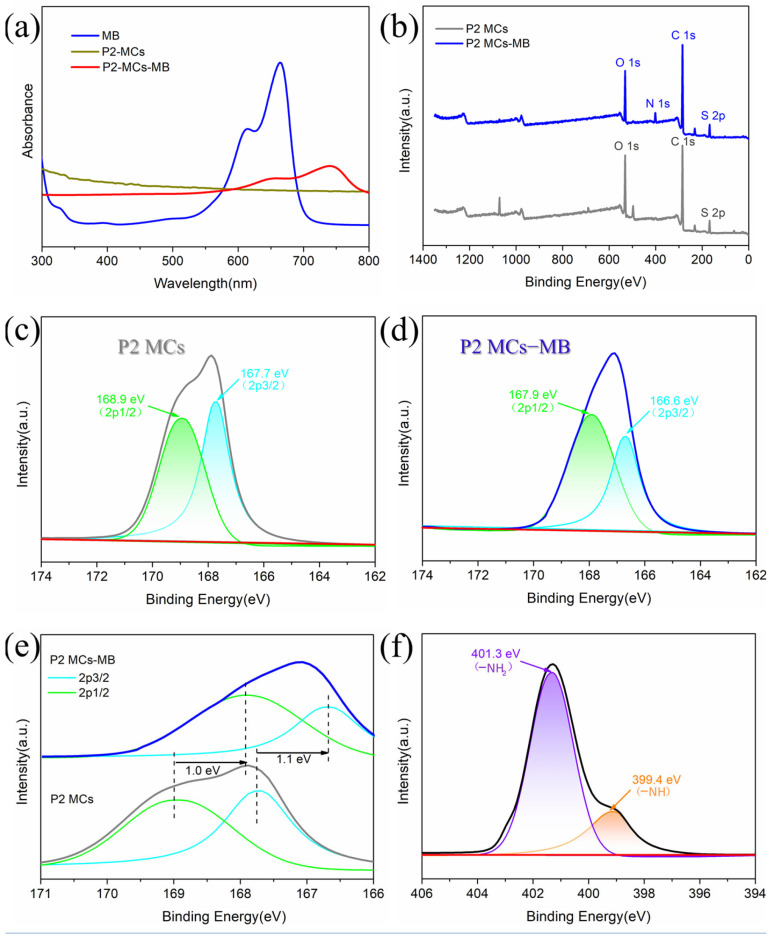
(**a**) UV spectra of MB, P2-MCs, and P2-MCs–MB, (**b**) XPS spectra of P2-MCs, and P2-MCs–MB, (**c**,**d**) Peak-fitting XPS spectra of P2-MCs, and P2-MCs–MB: S 2p regions, (**e**) Comparison of XPS spectra of S 2p regions before and after adsorption by P2-MCs, (**f**) XPS spectra of P2-MCs–MB: N 1s regions.

To study the adsorption mechanism of microcapsules for dyes, we measured UV–visible absorption spectra of MB, P2 MCs and P2 MCs–MB, respectively. It can be seen from Figure 9a that the characteristic peak of MB appears on the spectrum of P2 MCs–MB. This indicates that MB was adsorbed on P2 MCs. Compared with the UV–visible absorption spectra of MB, the characteristic absorption peak of MB in P2 MCs–MB spectrum has a significant red shift. This may be the interaction between P2 MCs and MB [47]. At the same time, the analysis results are consistent with the deduction of the cyclic adsorption experiment.

Figure 9b shows comparison of XPS spectra before and after MB adsorption by P2-MCs. The neonatal S 2p and N1s peaks appear in the spectrum of P2-MCs–MB compared with pure P2-MCs. It suggests MB molecules were adsorbed on P2-MCs. High-resolution spectra assigned to the S 2p core level peaks are de-convoluted using Gaussian fitting and presented in Figure 9c,d, respectively. Chemical states of -SO_3_ groups were detected by S 2p spectra of PSS in P2 microcapsules. The S 2p lines were fitted to a spin-split doublet, 2p3/2 and 2p1/2, of energy difference of 1.2 eV. In the P2 microcapsules’ spectrum, the 2p3/2 and 2p1/2 peaks are located at 167.7 and 168.9 eV, respectively [59] (Figure 9c). Upon adsorption of MB, In the S 2p spectrum (Figure 9d), sub-bands at 166.6 and 167.9 eV correspond to 2p3/2 and 2p1/2 peaks are assigned to 2p3/2 and 2p1/2 peaks of P2-MCs–MB, respectively. In Figure 9e, S 2p binding energies are decreased by about 1.1 eV. 

The N 1s spectra of P2 MCs–MB is shown in Figure 9f. For pure hollow P2–MB micro-particles, the N 1s spectra could be fit well with two different N bonds. Peaks at about 401.3 and 399.4 eV were corresponding to N in the groups of –NH_2_ and –NH, respectively [58]. 

### 2.6. The Secondary Adsorption of P2 and P2P Microcapsules to RhB and DY5G

**Figure 10:**
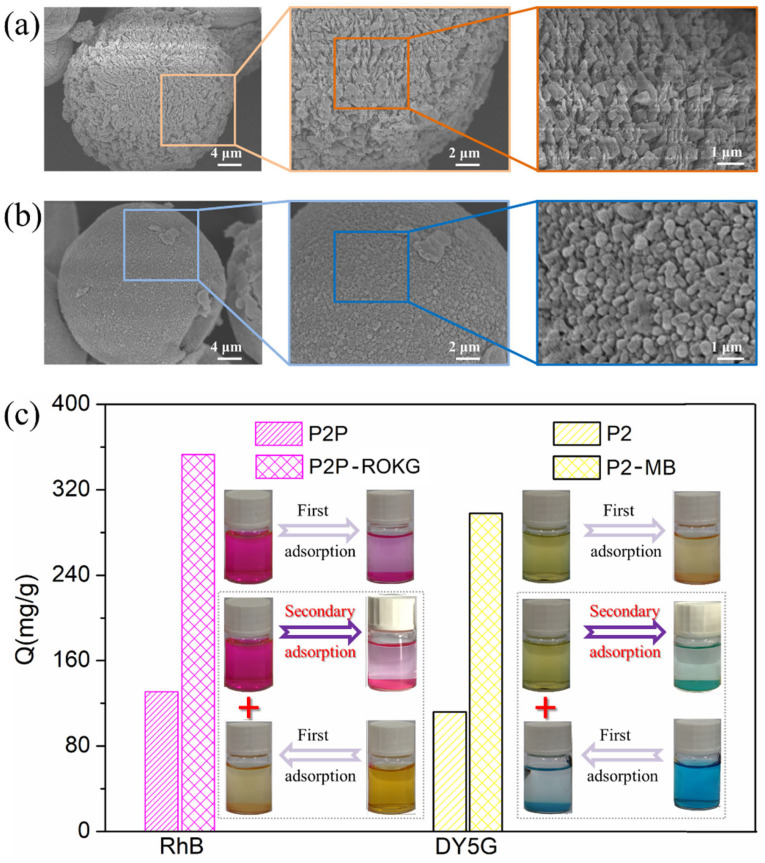
(**a**,**b**) SEM images of the P2P–ROKG and P2–MB micro-particles, (**c**) The adsorption of P2P–ROKG and P2–MB micro-particles to RB and DY5G.

Industrial wastewater often contains many kinds of contaminants. The capability of microcapsules to simultaneously treat cationic and anionic dye contaminants in wastewater should be determined. In Figure 10a,b, the P2P–ROKG and P2–MB micro-particles had a rough surface with a porous structure. Due to electrical properties of the dyes from the initial dye adsorption, many adsorption sites were provided for dyes, endowing P2P–ROKG and P2–MB micro-particles high secondary adsorption capacity. As seen in Figure 10c, P2P–ROKG and P2 –MB micro-particles that were obtained after the first adsorption can adsorb RhB and DY5G via secondary adsorption. However, the adsorption of the P2P–ROKG and P2–MB micro-particles for RhB and DY5G increased, reaching 352.56 mg/g and 297.69 mg/g. A large number of anionic dyes were distributed on the surface and pores of P2P–ROKG particles, which also enhanced the attraction of P2P–ROKG particles to RhB dye ions; on the other hand, the P2–MB micro-particles could adsorb DY5G (anionic dye), and these P2–MB micro-particles easily adsorbed anionic dyes for the second time through electrostatic attraction. P2P–ROKG and P2–MB particles adsorbed with anionic and cationic dyes can also be applied for secondary adsorption treatment of cationic and anionic dye wastewater, indicating the application potential of the two microcapsules in treatment of printing and dyeing wastewater.

### 2.7. Desorption

**Figure 11:**
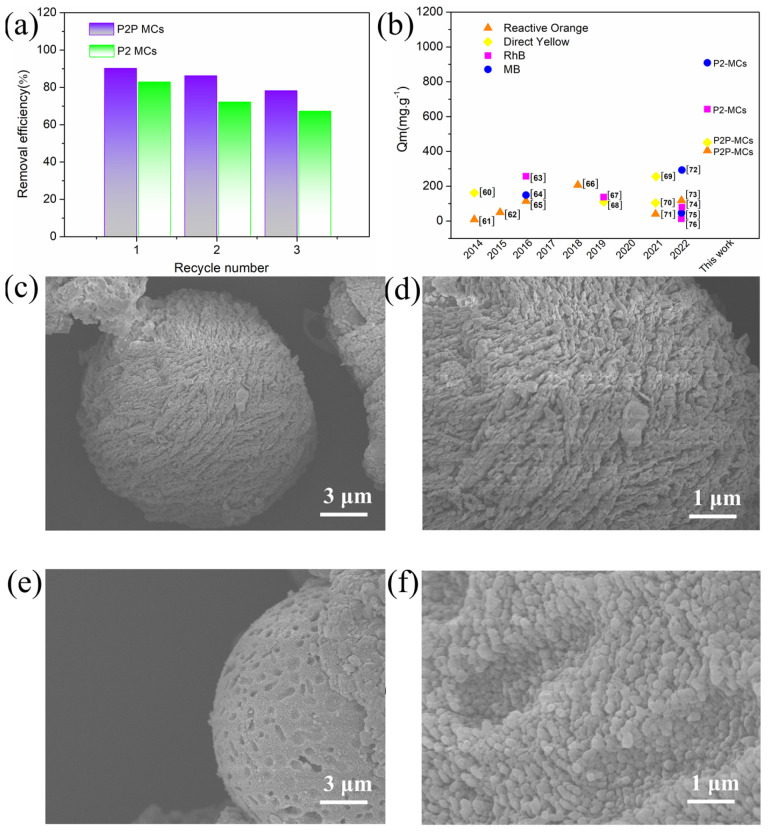
(**a**) Dye removal rate after three cycles of adsorption and desorption, (**b**) Comparison of some recently reported adsorbents with high maximum adsorption capacities to reactive orange, direct yellow, RhB and MB [60,61,62,63,64,65,66,67,68,69,70,71,72,73,74,75,76]. (**c**,**d**) SEM images of the P2P–ROKG micro-particles after three cycles of adsorption and desorption, (**e**,**f**) SEM images of the P2–MB micro-particles after three cycles of adsorption and desorption.

If strong bases or acids could remove dye molecules, it could also be clarified that dyes attach to adsorbent mainly through electrostatic interaction [51]. After P2P microcapsules adsorb ROKG dyes, the ROKG dyes could be released by soaking P2P microcapsules in 75% ethanol containing 1 M NaOH. After three cycles, the P2P microcapsule adsorbents maintain better removal efficiencies, decrease from 90.28% to 86.26% and 78.23%, which are shown in Figure 11a, respectively. Due to the different adsorption mechanisms, P2 microcapsules loaded with MB are mainly desorbed and regenerated with 75% ethanol and 1 M HCl mixed solution [7]. The R of MB were 82.37% to 71.66% after two cycles. However, P2 microcapsules still have a certain degree of reusability, and the removal efficiency of MB dyes was maintained at about 66.73% after three analyses.

Figure 11b compares the adsorption capacity of P2P and P2 microcapsules with other dye adsorbents reported previously [60,61,62,63,64,65,66,67,68,69,70,71,72,73,74,75,76]. Clearly, both P2P and P2 microcapsules display the highest adsorption capacities for ROKG, DY5G, RhB and MB. The superiority of P2P and P2 microcapsules and their potential application in highly-efficient and rapid removal of anionic and cationic dyes from water is shown in Figure 11b.

The cycle stability of P2P and P2 microcapsules is poor, which may be because after several adsorption and desorption cycles, the surface of the microcapsules have many adsorption sites and space is covered by adsorbed dye molecules, resulting in poor cycle performance. As shown in the SEM (Figure 11c–f), after three adsorption and desorption tests, the space on the surface of the microcapsules is filled with residual dye molecules.

For the desorbed strong acid and alkali solutions, in order to avoid causing pollution to the environment, we suggest that after desorption treatment, the strong acid and alkali waste liquid be collected in a centralized manner, and then neutralized according to the wastewater treatment principle of treating water with waste [77,78,79].

## 3. Materials and Methods

### 3.1. Materials

Ethylenediaminetetraacetic acid (EDTA) was bought from Beijing Puboxin Biotechnology Co., Ltd. Poly(allylamine hydrochloride) (PAH) and poly(styrene sulfonic acid) sodium salt (PSS M*_w_* ≈ 70 kDa) were purchased from Alfa Aesar (Tianjin) Co., Ltd. (Tianjin, China) Direct yellow 5G (DY5G), reactive orange K-G (ROKG), Rhodamine B (RhB) and methylene blue (MB) were bought from Shanghai ANOKY Group Co., Ltd. and their basic data are shown in Table 5.

### 3.2. Preparation of (PAH/PSS)_2_ and (PAH/PSS)_2_PAH

In brief, Ca(NO_3_)_2_·4H_2_O (100 mL, 0.025 M) solution consisting of PSS (0.20 g, M*_w_* ≈ 70 kDa) was poured into Na_2_CO_3_ (0.025 M) under magnetic stirring. After maintaining for 25~30 min, the CaCO_3_ templates were washed and collected.

**Scheme 1:**
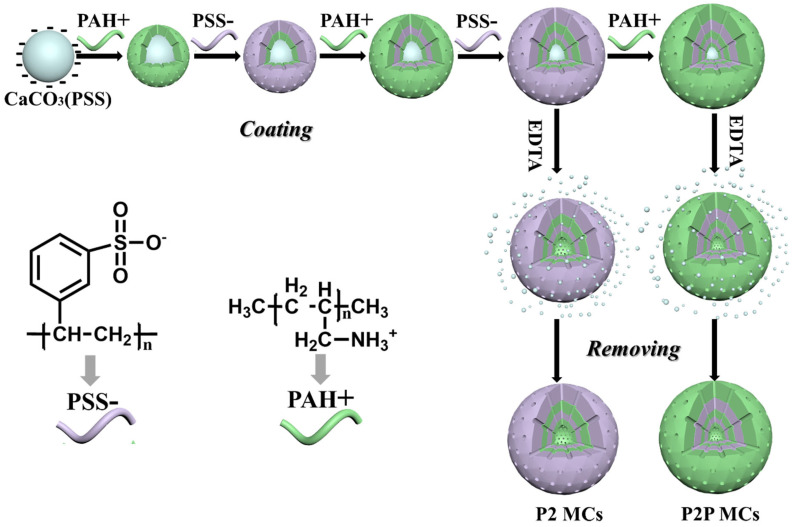
The schematic process of preparing polyelectrolyte P2P and P2 microcapsules.

CaCO_3_ templates were dispersed into PAH solution (10 mL, 0.1 mg/mL, 0.5 M NaCl) for 20 min under continuous shaking (100–120 rpm/min). Then, the coated particles were incubated in 10 mL PSS solution (0.1 mg/mL, 0.5 M NaCl) using the same procedure. The multilayer structure was formed by alternative assembly of corresponding materials for four or five times ((PAH/PSS)_2_ as P2 microcapsules or (PAH/PSS)_2_PAH as P2P microcapsules). The hollow P2 and P2P microcapsules were obtained through etching CaCO_3_ templates by using 30 mL EDTA solution (0.1 M, pH 7.0). The process is shown in Scheme 1.

### 3.3. Characterization of (PAH/PSS)_n_PAH Microcapsules

The P2 or P2P microcapsules after drying, were studied with SEM (Hitachi S4800, HITACHI) under a 20 kV accelerating voltage and the diluted P2 or P2P microcapsules solution was dropped onto a 400-mesh TEM carbon coated copper grid to prepare for TEM (Hitachi H7650, HITACHI) analysis of the sample. FTIR was tested on a Thermo Scientific Nicolet iS20 spectrometer. Particle size of PSS-doped CaCO_3_ particles were analyzed by a HORIBA LA-300 Laser Diffraction Analyzer. The microcapsule samples were freeze-dried for thermogravimetric analysis. The thermogravimetric analyzer STA 409PC (NETZSCH Scientific Instruments Trading Ltd.) was used for detection. X-ray photoelectron spectroscopy (XPS) was analyzed by Thermo Scientific K-Alpha.

### 3.4. Adsorption Experiments

The adsorption experiments with P2 or P2P microcapsules were studied under different conditions. A UV-1200 spectrophotometer (Mapada Instrument Co., Ltd.) was used to test the absorbance of dyes embedded in P2 or P2P microcapsules. P2 and P2P microcapsules (1.0 × 10^8^) were placed in dye solution (200, 500, 1000, 2000, 2500 and 3000 mg/L) at pH = 4, 5, 6, 7, 8 and 9. Desorption of P2 and P2P microcapsule adsorbed dyes was studied by a methanol acetate mixed solution with a volume fraction of 10%. The adsorption capacity of P2 and P2P microcapsules to dyes under different conditions and the dye removal efficiency in the mixture of acetic acid and methanol (%) were calculated according to the following Equations (6) [80] and (5) [81].

(6) $$Q=\frac{{(C}_{0}-C_{e})V}{m}$$

(7) $$R=\frac{C_{0}-C_{t}}{C_{0}}\times 100$$

where Q (mg/g) is the mass of dye adsorbed by the adsorbent of unit mass. *C*_0_ (mg/L) and *C_e_* (mg/L) are the initial concentration of dye solution and residual dye concentration at adsorption equilibrium. *C_t_* (mg/L) represents the concentration of the mixed solution with change of time t after adding adsorbent, and *V* (L) represents the volume of dye solution. Additionally, *m* (g) is the mass of microcapsules. The dye removal efficiency (R, %) is the percentage of dye desorbed by the desorption agent.

## 4. Conclusions

In this study, the two P2P and P2 MCs with excellent adsorption performance for cationic dyes and anionic dyes were obtained by a layer-by-layer method. The resulting P2P microcapsules and P2 microcapsules have hollow structures and porous channels in their shells, which allow them to exhibit high adsorption capacity, a fast adsorption rate and good adsorption selectivity for different kinds of dyes. The P2 MCs show high adsorption capacities for RhB (642.26 mg/g) and methylene blue (909.25 mg/g), with an extremely low equilibrium adsorption time (~20 min).The P2P MCs exhibited high adsorption capacities of 404.79 and 451.56 mg/g for reactive orange K-G (ROKG) and direct yellow 5G (DY5G), respectively. P2P and P2 MCs’ experimental adsorption data fit the Langmuir isotherm model and pseudo-second-order model well. The excellent adsorption performance of P2P and P2 MCs on four dyes was mainly attributed to mechanisms of electrostatic interaction. The study might offer new ideas for the structure and design of new adsorbents with excellent performance.

## Figures and Tables

**Table 1:** Kinetic parameters for the adsorption of anionic dyes onto P2 and P2P microcapsules.

Cationic Dyes	Adsorbent	Pseudo-First-Order			Pseudo-Second-Order			Intraparticle Diffusion					
		Q_e,cal_ (mg/g)	k_1_ (min^−1^)	R_1_^2^	Q_e,cal_ (mg/g)	k_2_ (g·mg^−1^·min^−1^)	R_2_^2^	k_i1_ (mg·g^−1^·min^−1/2^)	c_1_ (mg/g)	R_i1_^2^	k_i2_ (mg·g^−1^·min^−1/2^)	c_2_ (mg/g)	R_i2_^2^
RhB	P2P	394.5180	0.0508	0.9243	426.0395	2.706 × 10^−4^	0.9988	53.3927	169.2147	0.8647	6.5780	316.3695	0.9218
	P2	600.0253	0.04833	0.5156	644.1718	1.206 × 10^−4^	0.9999	244.7164	7.5673	0.9371	0.9043	626.7337	0.7801
MB	P2P	459.6831	0.0305	0.8153	474.1180	2.705 × 10^−4^	0.9994	122.9298	114.1426	0.6103	3.6964	407.2721	0.9345
	P2	872.9607	0.0586	0.8588	912.4087	1.365 × 10^−4^	0.9995	96.1240	483.7519	0.9832	9.8377	740.1333	0.9676

**Table 2:** Kinetic parameters for the adsorption of cationic dyes onto P2P and P2 microcapsules.

Anionic Dyes	Adsorbent	Pseudo-First-Order			Pseudo-Second-Order			Intraparticle Diffusion					
		Q_e,cal_ (mg/g)	k_1_ (min^−1^)	R_1_^2^	Q_e,cal_ (mg/g)	k_2_ (g·mg^−1^·min^−1^)	R_2_^2^	k_i1_ (mg·g^−1^·min^−1/2^)	c_1_ (mg/g)	R_i1_^2^	k_i2_ (mg·g^−1^·min^−1/2^)	c_2_ (mg/g)	R_i2_^2^
ROKG	P2	210.1818	0.03552	0.87635	217.3913	7.0323 × 10^−4^	0.9985	12.4521	109.7804	0.8944	30.9230	238.1153	0.6772
	P2P	331.6909	0.01869	0.8822	384.0245	6.970 × 10^−5^	0.9771	25.9532	69.89	0.6226	16.3119	104.9623	0.9553
DY5G	P2	176.8772	0.0162	0.8979	188.75005	2.514 × 10^−4^	0.9858	30.7321	36.535	0.6652	1.8974	119.999	0.9615
	P2P	401.7688	0.0233	0.69724	450.5465	1.222 × 10^−4^	0.9853	8.8988	314.2024	0.8589	6.3555	321.5930	0.8005

**Table 3:** The isotherm constants and correlation coefficients of Langmuir and Freundlich models for the adsorption of ROKG and DY5G onto P2P and P2 microcapsules.

Dye	Adsorbent	Langmuir			Freundlich		
		qm	KL	R^2^	KF	n	R^2^
		(mg·g^−1^)	(L·g^−1^)		(mg·g^−1^)		
ROKG	P2P	478.4689	1.8993	0.9979	14.4877	2.4	0.9492
	P2	478.43	1.25	0.9939	6.519	1.9723	0.9767
DY5G	P2P	561.1672	1.2875	0.9949	7.8491	1.9743	0.966
	P2	433.0047	0.9277	0.9999	0.0311	0.8834	0.968

**Table 4:** The isotherm constants and correlation coefficients of Langmuir and Freundlich models for the adsorption of RhB and MB onto P2P and P2 microcapsules.

Dye	Adsorbent	Langmuir			Freundlich		
		qm	KL	R^2^	KF	n	R^2^
		(mg·g^−1^)	(L·g^−1^)		(mg·g^−1^)		
RhB	P2P	750.0694	1.0701	0.9989	7.7512	1.8677	0.9605
	P2	875.0628	0.8901	0.9982	0.1872	0.9835	0.9739
MB	P2P	1217.879	0.8335	0.9891	7.51927	1.6864	0.931
	P2	1239.065	0.895	0.9989	0.3124	1.004	0.9733

**Table 5:** Basic data of model dyes investigated in this work.

Dye	Abbreviation	λmax ^a^ (nm)	Molecular Formula	Molecular Structure	Category
**Reactive orange K-G**	ROKG	413	C_19_H_16_N_7_Na_3_O_10_S_3_Cl	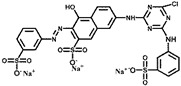	Anionic
**Direct yellow 5G**	DY5G	393	C_24_H_19_N_4_NaO_5_S_2_	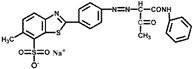	Anionic
**Rhodamine-B**	RhB	554	C_28_H_31_N_2_O_3_Cl	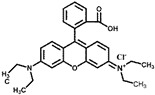	cationic
**Methylene Blue**	MB	664	C_16_H_18_ClN_3_S	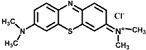	cationic

^a^ maximum absorption wavelength (nm).

## Data Availability

Not applicable to this article.

## Supplementary Materials

The following supporting information can be downloaded at: https://www.mdpi.com/article/10.3390/molecules28073010/s1, Figure S1: Optical images (**a**,**b**) and SEM (**c**,**d**) of the CaCO_3_ template; Figure S2: Optical images of the P2P and P2 microcapsules; Figure S3 TG-DTG spectra of CaCO_3_, P2P and P2 microcapsules.
