# Supplementary material for: Rapid, Highly-Efficient and Selective Removal of Anionic and Cationic Dyes from Wastewater Using Hollow Polyelectrolyte Microcapsules
Source: Molecules. 2023 Mar 28;28(7):3010. doi: 10.3390/molecules28073010 (PMC10095712; doi:10.3390/molecules28073010)
Supplement: Supplementary file 1 [file molecules-28-03010-s001.zip › molecules-2295762-supplementary.pdf]

Supporting Information

Rapid, Highly-Efficient and Selective Removal of Anionic and Cationic Dyes from Wastewater Using Hollow Polyelectrolyte Microcapsules

Zhiqi Zhao [1], Hongbing Zhou [2], Xu Han [1], Lun Han [1], Zhenzhen Xu [1,*] and Peng Wang [1,*]

[1] School of Textile and Garment, Anhui Polytechnic University, Wuhu 241000, China; zhaozhiqi@mail.ahpu.edu.cn (Z.Z.); hanxu@ahpu.edu.cn (X.H.); dayanqingwashou@163.com (L.H.)

[2] Zhejiang Huaguang Automotive Interior Decoration Co., Ltd., Rui'an 325200, China; 13958826789@139.com

* Correspondence: xuzhenzhen@ahpu.edu.cn (Z.X.); wangpeng@ahpu.edu.cn (P.W.)

Figure captions

Figure S1. Optical images (**a**,**b**) and SEM (**c**,**d**) of the $CaCO_3$ template.

Figure S2. Optical images of the P2P and P2 microcapsules.

Figure S3. TG-DTG spectra of $CaCO_3$, P2P and P2 microcapsules.

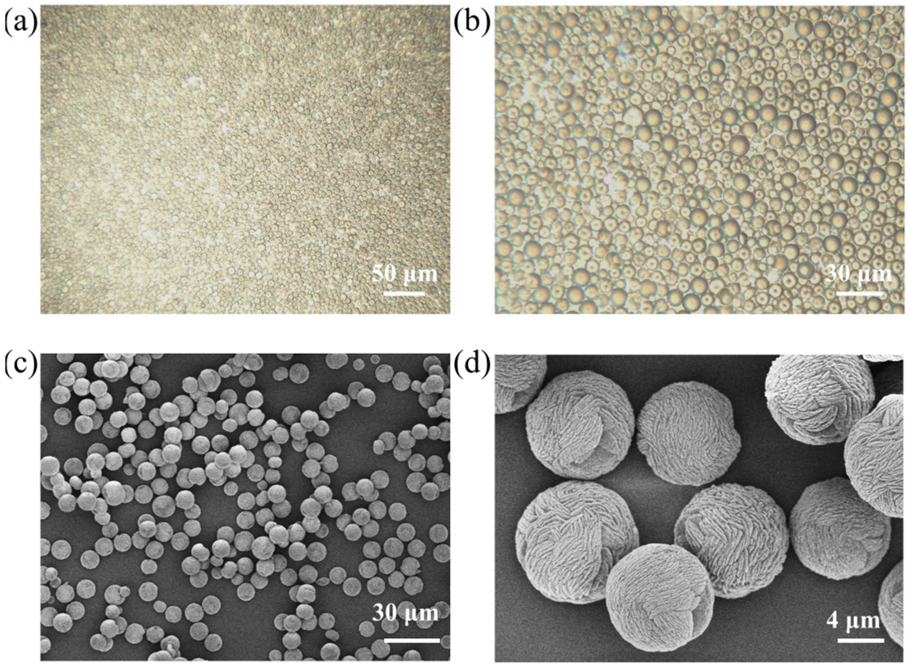

Figure S1.

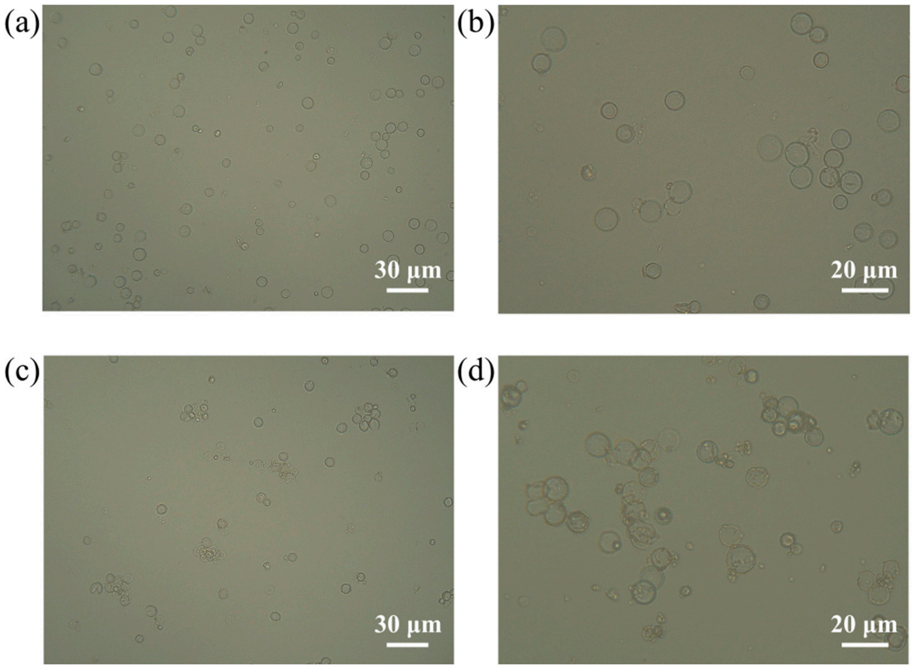

Figure S2.

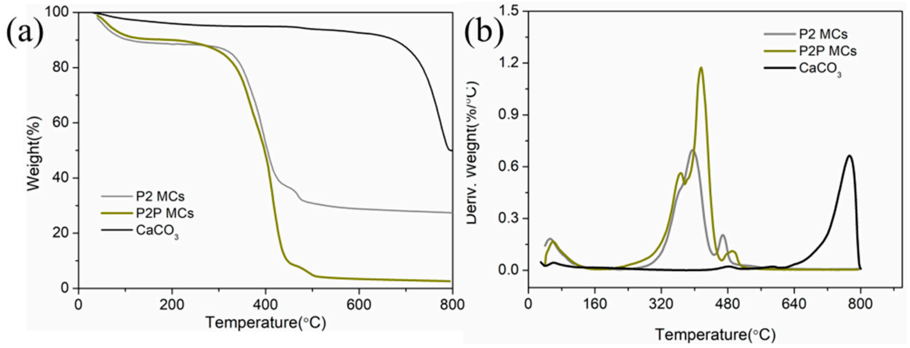

Figure S3.
